# Abnormalities in nitric oxide synthase system function and their association with cognitive function in first-episode schizophrenia patients

**DOI:** 10.1038/s41537-026-00764-9

**Published:** 2026-05-12

**Authors:** Fei Jiang, Jie Hou, Qun Yang, Peijuan Wang, Lei Ji, Jing Cao, Xiaoying Zhang, Qing Tian, Xiaobin Zhang

**Affiliations:** 1 Department of Psychiatry, Nantong Mental Health Center, The Fourth People’s Hospital of Nantong, Nantong, P. R. China; 2 The Affiliated Guangji Hospital of Soochow University, Suzhou, Jiangsu P. R. China

**Keywords:** Human behaviour, Biomarkers

## Abstract

Cognitive deficits are a core feature of schizophrenia, emerging early and strongly influencing functional outcomes. However, the role of the nitric oxide (NO) signaling pathway in drug-naïve, first-episode schizophrenia remains unclear. To investigate the relationship between the nitric oxide synthase (NOS) system and cognitive function, we studied 98 first-episode, drug-naïve schizophrenia patients and 96 matched healthy controls. Plasma levels of inducible NOS (iNOS), total NOS (TNOS), the iNOS/TNOS ratio, malondialdehyde (MDA), and hydrogen peroxide (H₂O₂) were measured. Psychopathological symptoms and cognitive performance were assessed using the Positive and Negative Syndrome Scale (PANSS) and the Repeatable Battery for the Assessment of Neuropsychological Status (RBANS), respectively. Group differences and associations among NOS markers, cognitive domains, and symptom severity were examined. Patients exhibited marked NOS system dysfunction, characterized by reduced iNOS and TNOS levels, a lower iNOS/TNOS ratio, elevated MDA levels, and reduced H₂O₂ concentrations. TNOS levels were positively associated with RBANS total score and multiple cognitive domains, independent of PANSS total score, whereas iNOS levels were associated only with immediate memory. The iNOS/TNOS ratio showed no independent association with cognition. In addition, TNOS levels were correlated with PANSS total score, suggesting that overall NOS function reflects disease burden. These findings indicate that early schizophrenia is characterized by NOS system dysfunction. Overall NOS activity, rather than relative NOS subtype expression, is closely linked to multidimensional cognitive impairment and is partly independent of symptom severity.

## Introduction

Schizophrenia (SCZ) is a chronic mental disorder primarily characterized by disturbances in cognition, emotion, and behavior^1^. Beyond positive and negative symptoms, cognitive impairment is widely recognized as one of the core features of SCZ, emerging even in its early stages^2^. Cognitive deficits not only markedly impair patients’ social functioning and quality of life but are also closely associated with poor long-term outcomes^3^. Moreover, existing antipsychotic drugs have limited cognitive-enhancing effects^4^. Therefore, it is crucial to elucidate the biological mechanisms underlying cognitive impairment in SCZ to better understand the nature of the disease and identify novel intervention targets.

In recent years, the role of oxidative stress in the pathogenesis of SCZ has received widespread attention^5^. Nitric oxide (NO) is a crucial signaling molecule that participates in key processes in the central nervous system (CNS), including neurotransmitter release, regulation of synaptic plasticity, and neurovascular coupling^6^. NO production is catalyzed by nitric oxide synthase (NOS), where inducible NOS (iNOS) is typically activated during inflammation or stress, yielding a higher level of NO^7^. Total nitric oxide synthase (TNOS) represents a composite measure of nitric oxide synthase enzymatic activity detectable in biological samples, reflecting the combined activity of NOS isoforms that contribute to systemic nitric oxide production rather than a direct indicator of NOS signaling activity within specific tissues^8^. Previous studies suggest that evaluating nitric oxide signaling from a system-level biochemical perspective, rather than focusing exclusively on individual NOS isoforms, may help characterize alterations in NO-related pathways involved in inflammatory and physiological processes^9^. Based on this concept, the iNOS/TNOS ratio is regarded as a comprehensive indicator that reflects the degree of iNOS activation relative to the overall NOS functional state. This ratio has been used in research on inflammatory diseases and neuroscience-related studies to characterize the functional imbalance in NO signaling^8^. However, the relationship between this indicator and cognitive function in SCZ, particularly among first-episode SCZ patients, remains understudied. Abnormalities in the NO signaling pathway may impair neuronal function through oxidative/nitrosative stress mechanisms, further affecting neural processes related to cognitive processing^10^.

Previous studies have examined the relationship between the NOS system and SCZ from various perspectives. For instance, some studies reported alterations in peripheral or central NO-related markers among SCZ patients. However, conclusions regarding the direction of these changes and their clinical significance remain inconsistent across different studies^11^. In addition, some studies suggest that NOS-related abnormalities may be associated with symptom severity or cognitive function; however, these findings are predominantly observed in chronic SCZ patients or those without systematic control of medication factors, limiting the comparability of results and the interpretation of underlying mechanisms^12,13^. Compared to chronic SCZ patients, individuals with first-episode SCZ are less affected by long-term disease progression and continuous medication treatment, making them more conducive to reflect disease-related intrinsic biological abnormalities^14^. In addition, the NO signaling pathway is closely associated with oxidative stress processes. Malondialdehyde (MDA), a lipid peroxidation product, and hydrogen peroxide (H₂O₂), a reactive oxygen species, are commonly used as downstream oxidative damage markers related to the NO signaling pathway^15,16^. However, there is a lack of studies exclusively based on systematic integration of cognitive function assessment with changes in the NOS system. This limitation has hindered our understanding of how NO-related mechanisms influence cognitive impairments associated with SCZ^2^.

Therefore, the present study included first-episode SCZ patients to examine changes in the plasma levels of iNOS, TNOS, iNOS/TNOS ratio, and related oxidative stress markers (MDA and H₂O₂) and to analyze the relationship between these indicators and cognitive function. We hypothesized that in the early disease stage, the overall functional state of the NOS system may better reflect the biological mechanisms associated with cognitive impairment compared to the NOS subtype ratio and that this association is, to some extent, independent of overall symptom severity.

## Material and methods

### Participants

This study consecutively enrolled 98 patients with first-episode SCZ who were hospitalized for the first time in the Department of Psychiatry at Nantong Fourth People’s Hospital between March 2022 and November 2024 and had not received treatment with antipsychotic medications. The inclusion criteria were as follows: (1) meeting the diagnostic criteria for SCZ according to the Diagnostic and Statistical Manual of Mental Disorders, Fifth Edition (DSM-5)^17^ and confirmed to have SCZ based on an assessment using the Chinese version of the Structured Clinical Interview for DSM-IV Disorders (SCID-I/P); (2) elementary school or higher level of education, with the ability to complete cognitive function assessments; (3) first-episode cases with no prior history of systemic treatment with antipsychotic medications; (4) diagnosis confirmed by two psychiatrists with attending physician qualifications; (5) age between 18 and 65 years; and (6) voluntary participation in the study with written informed consent provided by the patient.

The exclusion criteria were (1) concurrent diagnosis of a defined organic brain disorder; (2) history of past or current psychoactive substance abuse or dependence; (3) presence of severe physical illness; (4) pregnancy or lactation; and (5) receipt of modified electroconvulsive therapy during the study period or in the past.

During the study period, 96 healthy controls were recruited based on matching principles for age, gender, and years of education. The inclusion criteria for the control group were (1) age 18–65 years with elementary school or higher level of education; (2) no history of mental disorders as assessed by the Chinese version of the Mini-International Dementia Interview (MINI); (3) no history of major mental disorders among first-degree relatives or collateral relatives within three generations; (4) no history of neurological disorders; (5) no concomitant severe physical illnesses; (6) no history of psychoactive substance use or drug dependence; (7) no pregnancy or breastfeeding for female participants; and (8) voluntary participation with signed written informed consent.

Prior to study implementation, all participants or their legal guardians were fully informed of the study’s objectives, procedures, and potential risks. Written informed consent was obtained on a strictly voluntary basis. All research data were anonymized to protect participant privacy. The study was conducted in accordance with the ethical principles of the Declaration of Helsinki and local institutional guidelines. The study protocol was reviewed and approved by the Ethics Committee of Nantong Fourth People’s Hospital (Ethics Approval No.: 2022-K015).

### Clinical and cognitive assessment

Study data were mainly collected through the following two pathways: (1) a questionnaire designed by the research team was used to gather general demographic and clinical information from both patient and healthy control groups. This information included age, gender, height, weight, body mass index (BMI), educational level, family history of mental illness, and smoking status; (2) In addition, disease-related information, such as age at onset and disease duration, was obtained from the patient group.

Psychiatric symptoms were systematically assessed using the Positive and Negative Syndrome Scale (PANSS)^18^. This scale comprises 30 items assessing positive symptoms (7 items), negative symptoms (7 items), and general psychopathology symptoms (16 items). Three supplementary items are also included for assessing aggression risk but are excluded from total score calculation. The response to each item is based on a 7-point rating scale (1 indicates no symptoms, 7 indicates extremely severe symptoms). The PANSS total score ranges from 30 to 210 points, with higher scores indicating greater severity of psychotic symptoms.

The Repeatable Battery for the Assessment of Neuropsychological Status (RBANS) was employed to evaluate participants’ cognitive function. The assessment was administered by psychiatrists who received standardized training and was conducted after patients completed diagnostic evaluations and were in a relatively stable condition. The RBANS offers advantages such as a simple administration process, relatively short assessment time, and the ability for a single rater to complete it independently, making it widely used in SCZ-related research. The RBANS encompasses five cognitive domains: immediate memory, visuospatial/constructional abilities, language, attention, and delayed memory. It includes 12 subtests: Word List Learning, Immediate Story Retelling, Figure Copying, Line Orientation, Picture Naming, Verbal Fluency, Digit Span, Coding, Delayed Word List Recall, Word List Recognition, Delayed Story Retelling, and Delayed Figure Retrieval. Scores for each dimension and the total scale score reflect the corresponding cognitive domains and overall cognitive functioning. A higher total score indicates better overall cognitive functioning. Previous studies have confirmed that the RBANS has good reliability and validity in individuals with SCZ^19^. The present study strictly followed standardized scoring procedures, wherein raw scores from each subtest were converted to scale scores before incorporating them into statistical analysis.

### Fasting blood sample collection and biochemical analysis

Blood samples were collected from the patient group on an empty stomach the morning after their enrollment and before receiving any medication. For the healthy control group, a single blood sample was collected on an empty stomach during the baseline assessment phase. All blood collection procedures followed a standardized protocol: sampling was conducted between 07:00 and 09:00 AM, with 5 mL whole blood drawn from the antecubital vein into EDTA anticoagulant-coated tubes. Immediately after collection, the samples were centrifuged at 3000 rpm for 15 min at 4°C to separate plasma. Each plasma sample was sequentially numbered according to the collection order and aliquoted into four 0.5 mL cryovials. The samples were then uniformly transferred to −80 °C ultra-low temperature freezers for storage until subsequent centralized analysis.

Oxidative stress-related markers were strictly measured according to the kit instructions, with testing conducted by the Nanjing Jiancheng Biological Engineering Research Institute. TNOS and iNOS levels were quantified using a colorimetric assay kit (Catalog No.: A014-1). MDA concentration was measured using the thiobarbituric acid method by detecting the colored product formed by the reaction between MDA and thiobarbituric acid (Catalog No.: A003-1). H_2_O_2_ concentration was estimated by spectrophotometry (Catalog No.: A064-1-1). All samples were tested under identical conditions using kits from the same batch.

### Statistical analysis

Statistical analyses were performed using SPSS 26.0 (IBM, Chicago, IL, USA). Sample size was estimated using G*Power 3.1.9.7 (http://www.ats.ucla.edu/stat/gpower/). For comparisons of mean values, tests for two independent samples were applied, with statistical power set at 90%, significance level α = 0.10, and effect size set at 0.8. The Shapiro–Wilk test and distribution plots were used to assess the normality of continuous variables. Normally distributed continuous variables were expressed as mean ± standard deviation (SD), with intergroup comparisons performed using Student’s t test. Non-normally distributed continuous variables were reported as median (25th–75th percentile). Categorical variables were analyzed using the chi-square test. Biological markers exhibiting non-normal distribution (iNOS, MDA, and H₂O₂ levels) were subjected to natural logarithmic transformation to meet the statistical assumptions for subsequent parametric tests. Statistical power analysis was conducted during the study design phase to ensure sufficient sample size for detecting large effect differences (Cohen’s d ≥ 0.8). Effect sizes were reported using Cohen’s d, where the values of 0.2, 0.5, and 0.8 indicate a small, moderate, and large effect, respectively. Statistical significance was set at *p* < 0.05.

Differences in oxidative stress markers between the groups were initially assessed using multivariate analysis of covariance (MANCOVA). The dependent variables included iNOS and TNOS levels, iNOS/TNOS ratio, and MDA and H₂O₂ concentrations. The diagnostic group (patients vs. healthy controls) served as the fixed factor, with age, gender, years of education, BMI, and smoking status incorporated as covariates. Prior to the analysis, model assumptions were tested and found to meet analytical requirements. Subsequently, univariate analysis of covariance (ANCOVA) was performed for each indicator by using the same covariates mentioned above, with multiple comparisons adjusted using Bonferroni correction.

Pearson’s correlation analysis was used for normally distributed data, while Spearman’s correlation analysis was utilized for non-normally distributed data. To control for multiple comparison errors, Bonferroni correction was applied to correlations between oxidative stress markers and clinical/cognitive scores: for comparison between the PANSS total score and three subscale scores, the significance threshold was set at *p* < 0.0125 (0.05/4); for comparison between the RBANS total score and five cognitive subscale scores, the significance threshold was set at *p* < 0.0083 (0.05/6).

Finally, exploratory multiple regression analyses were conducted to examine the independent associations between oxidative stress markers (iNOS, TNOS, iNOS/TNOS ratio, MDA, and H₂O₂) and PANSS and RBANS total scores and subscale scores after adjustment for demographic covariates. These models therefore estimate adjusted associations between NOS-related biomarkers and clinical or cognitive variables rather than simple correlations. All statistical tests were two-tailed, with statistical significance set at *p* < 0.05.

## Results

### Comparison of demographic and general clinical data

Table 1 summarizes the demographic characteristics and clinical data of the SCZ patient and healthy control groups. The two groups showed no statistically significant differences in age, gender, years of education, BMI, and smoking status (all *p* > 0.05). The mean disease duration in the patient group was 6.57 ± 9.17 months. In the PANSS assessment, the mean values of positive symptom scores, negative symptom scores, general psychopathology scores, and total scores were 21.57 ± 4.37, 19.51 ± 4.88, 44.40 ± 3.57, and 85.48 ± 5.79, respectively.

**Table 1:** Demographic data of SCZ patients and healthy controls (mean ± SD).

	Patients (n = 98)	Controls(n = 96)	*t/z/χ*^*2*^	*P*
Age (years)	32(25,41)	33(23,41)	-0.651^b^	0.515
Gender (male/female)	36/62	31/65	0.423^c^	0.515
Education (years)	16(12,16)	15(13,16)	-0.775^b^	0.438
BMI (kg/m^2^)	21.81 ± 3.45	21.69 ± 3.10	0.254^a^	0.800
Smoking (yes/no)	7/91	8/88	0.096^c^	0.756
Duration of illness (months)	6.57 ± 9.17	–	–	–
P subscore	21.57 ± 4.37	–	–	–
N subscore	19.51 ± 4.88	–	–	–
G subscore	44.40 ± 3.57	–	–	–
PANSS total score	85.48 ± 5.79	–	–	–

*BMI* body mass index, *PANSS* Positive and Negative Symptom Scale, *P* PANSS positive symptom subscale, *N* PANSS negative symptom subscale, *G* PANSS general psychopathology subscale.

^a^Independent samples t test.

^b^Mann–Whitney *U* test.

^c^χ^2^ test.

### Levels of oxidative stress markers between SCZ patients and healthy controls

MANCOVA revealed significant between-group differences in the overall oxidative stress profile between the patient and healthy control groups (*F* = 50.957, *p* < 0.001). Subsequent ANCOVA, controlling for age, gender, education level, BMI, and smoking status, revealed significant differences between the patient and healthy control groups in the plasma levels of multiple oxidative stress markers (Table 2). Specifically, the patient group exhibited a significantly higher MDA level (*F* = 10.087, *p* = 0.002) than the control group, while iNOS level (*F* = 195.794, *p* < 0.001), TNOS level (*F* = 80.023, *p* < 0.001), iNOS/TNOS ratio (*F* = 126.495, *p* < 0.001), and H₂O₂ level (*F* = 6.144, *p* = 0.014) were significantly lower in the patient group than in the control group.

**Table 2:** Levels of oxidative stress markers between SCZ patients and healthy controls.

	Patients (n = 98)	Controls (n = 96)	*F*^a^	*p*
iNOS^△^(U/mL)	0.68 ± 0.14	0.89 ± 0.06	195.794	0.000
TNOS(U/mL)	9.90 ± 1.91	12.05 ± 1.43	80.023	0.000
iNOS/TNOS ratio	0.51 ± 0.12	0.66 ± 0.07	126.495	0.000
MDA^△^(nmol/ml)	0.42 ± 0.21	0.33 ± 0.18	10.087	0.002
H₂O₂^△^(mmol/L)	1.62 ± 0.23	1.67 ± 0.18	6.144	0.014

△, the result of natural logarithmic transformation.

*iNOS* inducible nitric oxide synthase, *TNOS* total nitric oxide synthase, *MDA* malondialdehyde, *H₂O₂* hydrogen peroxide.

^a^after controlling for age, sex, education, BMI, and smoking status, the differences in iNOS, TNOS, iNOS/TNOS ratio, MDA, H₂O₂ remained significant.

### Comparison of two sets of cognitive functions

The RBANS total scores and individual cognitive subscale scores were compared between the patient and healthy control groups (Table 3). MANCOVA revealed significant between-group differences across the overall cognitive spectrum (*F* = 118.428, *p* < 0.001). Subsequent ANCOVA, controlling for age, gender, education level, BMI, and smoking status, revealed that the patient group scored significantly lower than the healthy control group on the RBANS total score (*F* = 271.038, *p* < 0.001), immediate memory (*F* = 44.440, *p* < 0.001), visuospatial/constructional abilities (*F* = 296.973, *p* < 0.001), language function (*F* = 8.898, *p* < 0.01), attention (*F* = 445.618, *p* < 0.001), and delayed memory (*F* = 197.134, *p* < 0.001). Overall, the RBANS total score and all cognitive subscale scores were significantly lower in the patient group than in the healthy control group (all *p* < 0.01).

**Table 3:** Total and index scores on the RBANS in SCZ patients vs. healthy controls.

Index	patients (n = 98)	controls(n = 96)	*F*^a^	*p*
Immediate memory	1.90 ± 0.07	1.96 ± 0.07	44.440	0.000***
Visuospatial/constructional	1.86 ± 0.07	2.01 ± 0.06	296.973	0.000***
Language	1.98 ± 0.04	2.00 ± 0.05	8.898	0.003**
Attention	1.87 ± 0.08	2.05 ± 0.05	445.618	0.000***
Delayed memory	1.83 ± 0.11	1.99 ± 0.06	197.134	0.000***
Total	1.86 ± 0.08	2.00 ± 0.06	271.038	0.000***

**, *p* < 0.01; ***, *p* < 0.001. RBANS, Repeatable Battery for the Assessment of Neuropsychological Status. △, the result of natural logarithmic transformation.

^a^F value was controlled for age, smoking status, BMI, and education level.

### Correlation between the plasma levels of oxidative stress markers and psychiatric symptoms in the patient group

#### Correlation analyses

Correlation analysis was performed between the plasma levels of oxidative stress markers and PANSS total scores and subscale scores in the patient group, with the application of Bonferroni correction. The TNOS level showed a significant negative correlation with the PANSS total score (*r* = –0.210, *p* = 0.038, *p* corrected = 0.152) (Fig. S1). The remaining markers showed no significant correlations with the symptoms (all *p* > 0.05).

#### Regression analyses controlling for confounding factors

**Fig. 1:**
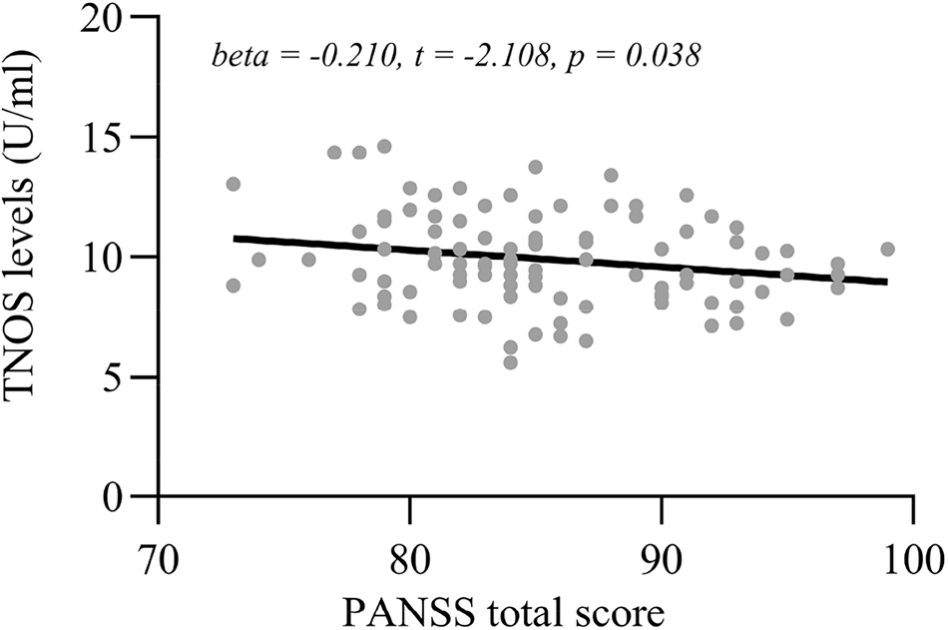
Correlations between TNOS levels and PANSS total scores. Scatter plot showing the correlation between plasma TNOS levels (U/ml) and PANSS total scores. TNOS, total nitric oxide synthase; PANSS, Positive and Negative Syndrome Scale.

Stepwise multiple regression analysis, controlling for age, gender, education level, BMI, smoking status, and disease duration as independent variables and potential confounders, revealed a significant correlation between the TNOS level and the PANSS total score (*beta* = −0.210, *t* = −2.108, *p* = 0.038) (Fig. 1).

### Correlation between the plasma levels of oxidative stress markers and cognitive function in the patient group

#### Correlation analyses

Correlation analysis was conducted between the plasma levels of oxidative stress markers and RBANS total scores and subscale scores in the patient group, with the application of Bonferroni correction. Significant positive correlations were observed between the iNOS level and immediate memory (*r* = 0.306), Visuospatial/constructional (*r* = 0.561), attention (*r* = 0.559), delayed memory (*r* = 0.475), and RBANS total score (*r* = 0.514) (Fig. S2). The TNOS level showed significant positive correlations with immediate memory (*r* = 0.286), visuospatial/constructional (*r* = 0.427), attention (*r* = 0.458), delayed memory (*r* = 0.338), and RBANS total score (*r* = 0.432) (*p* < 0.001, correlations remained significant after correction) (Fig. S3). The iNOS/TNOS ratio exhibited significant positive correlations with immediate memory (*r* = 0.207), visuospatial/constructional (*r* = 0.422), attention (*r* = 0.466), delayed memory (*r* = 0.390), and RBANS total score (*r* = 0.413) (*p* < 0.01; corrected *p* values showed non-significant correlation with immediate memory, while other correlations remained significant). The MDA level showed negative correlations with immediate memory (*r* = -0.173), visuospatial/constructional(*r* = -0.149), attention (*r* = -0.186), delayed memory (*r* = -0.148), and RBANS total score (*r* = -0.186) (*p* < 0.05; all correlations were non-significant after correction). The H_2_O_2_ level displayed a negative correlation with verbal scores (*r* = -0.161, *p* = 0.025, *p* corrected = 0.150).

#### Regression analyses with controlling for confounding factors

**Fig. 2:**
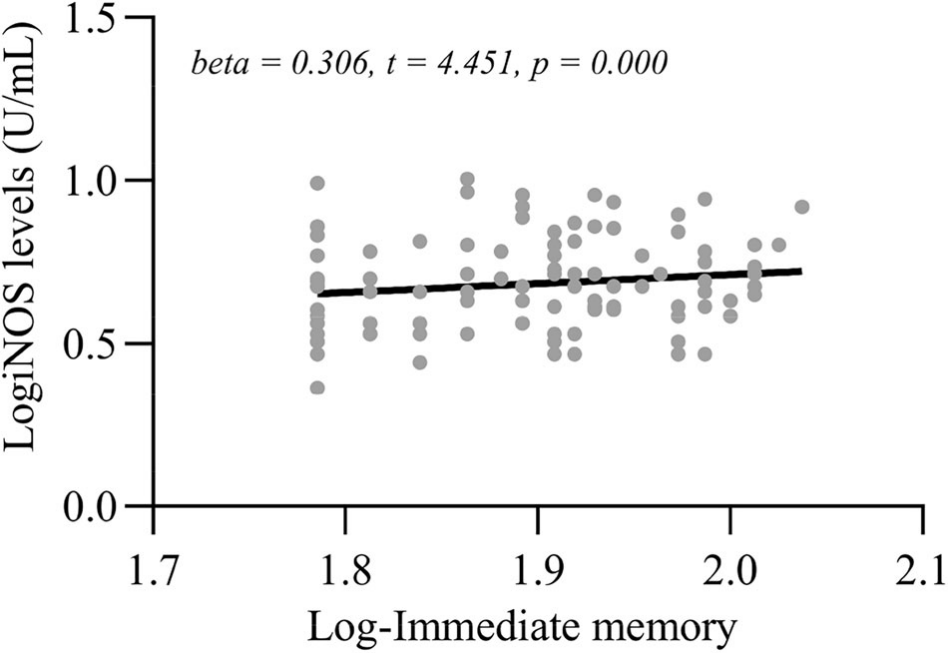
Correlations between log iNOS levels and immediate memory subscores. Scatter plot showing the correlation between plasma iNOS levels (U/mL) and immediate memory subscores.iNOS, inducible nitric oxide synthase.

**Fig. 3:**
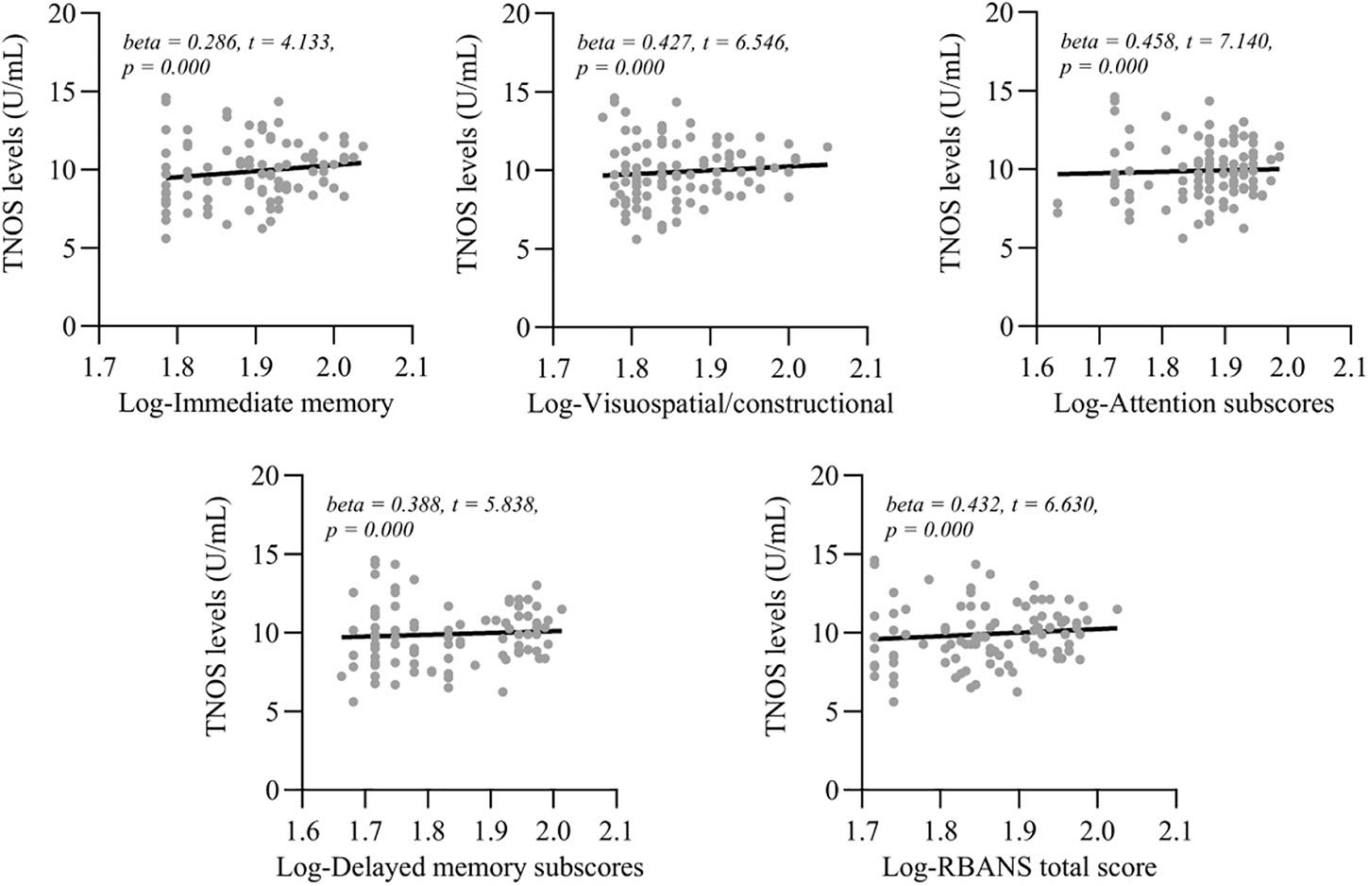
Correlations between TNOS levels and RBANS total and factor scores. Scatter plots showing the correlations between plasma TNOS levels (U/mL) and RBANS cognitive performance. TNOS, total nitric oxide synthase; RBANS,Repeatable Battery for the Assessment of Neuropsychological Status.

Stepwise multiple regression analysis, controlling for age, gender, education level, smoking status, and PANSS scores as independent variables and potential confounders, revealed a significant correlation between the iNOS level and immediate memory scores (*beta* = 0.306, *t* = 4.451, *p* = 0.000) (Fig. 2), while the TNOS level was significantly correlated with immediate memory scores (*beta* = 0.286, *t* = 4.133, *p* = 0.000) and visuospatial/constructional (*beta* = 0.427, *t* = 6.546, *p* = 0.000). The TNOS level also showed significant correlations with attention scores (*beta* = 0.458, *t* = 7.140, *p* = 0.000), delayed memory scores (*beta* = 0.388, *t* = 5.838, *p* = 0.000), and RBANS total scores (*beta* = 0.432, *t* = 6.630, *p* = 0.000) (Fig. 3).

## Discussion

The present study systematically investigated the relationship between oxidative stress markers and cognitive function in first-episode SCZ patients, with a focus on the NOS system. The following key findings were obtained: (1) First-episode SCZ patients exhibited dysfunction of the overall NOS system, characterized by reduced iNOS and TNOS levels and accompanied by elevated lipid peroxidation-related markers; (2) the TNOS level showed stable associations with overall cognitive function and multiple cognitive domains, and these associations remained significant after adjustment for demographic variables and overall symptom severity. In contrast, the iNOS level exhibited a specific association only with immediate memory; (3) In the multivariate regression analysis, the TNOS level was significantly correlated with PANSS total scores, indicating an association between overall NOS system function and the disease’s overall symptom burden; and (4) The iNOS/TNOS ratio exhibited no independent association with cognitive function after controlling for confounding factors. These findings suggest that, compared to subtype ratios, the overall functional state of the NOS system may better reflect cognitive impairment characteristics in first-episode SCZ patients.

The most remarkable finding of this study is the extensive and stable association between the TNOS level and multidimensional cognitive function. TNOS has been used in previous studies as a composite indicator of NOS-related enzymatic activity detectable in peripheral samples, which may be associated with systemic nitric oxide bioavailability rather than directly reflecting NOS signaling activity in specific organs or tissues^9^. Previous studies have demonstrated that NO is extensively involved in key processes in the CNS, including the regulation of synaptic plasticity, integration of neural network information, and coupling of cerebral blood flow. The long-term homeostasis of NO levels is crucial for maintaining higher cognitive functions^9,20–22^. The present study found that the TNOS level showed significant associations with RBANS total scores and multiple cognitive subdomains. These associations remained independent even after controlling for age, gender, years of education, and PANSS total scores. This finding suggests that variations in NOS-related biochemical markers may represent a biological dimension associated with cognitive performance that is partly independent of clinical symptom severity. However, this association does not imply a direct causal relationship, but rather likely reflects a steady-state coupling between the overall functional state of the NOS system and cognitive-related neural processes^23^.

In contrast, the relationship between the iNOS level and cognitive function exhibits distinct domain specificity, showing significant association only with immediate memory. Immediate memory relies on rapid information encoding and short-term retention processes, making it highly sensitive to synaptic transmission efficiency and neuronal excitability^24,25^. iNOS is typically induced under inflammatory or stress-related conditions and can lead to increased nitric oxide (NO) production. However, nitric oxide also serves important physiological functions in the central nervous system, including the modulation of synaptic plasticity, neurotransmitter release, and neural signaling involved in learning and memory processes^6,10^. Experimental studies suggest that moderate levels of NO signaling may facilitate synaptic plasticity and long-term potentiation, both of which are essential for memory formation and cognitive processing^22^. The resulting fluctuations in NO may more readily influence short-term neural information processing, without necessarily determining overall or long-term cognitive abilities^26^. A noteworthy observation is that this finding may differ from the broader association between iNOS and cognition observed in some previous studies involving patients with chronic SCZ^27^. Chronic SCZ patients typically experience persistent activation of the inflammatory state, cumulative oxidative stress, and prolonged drug exposure. Their abnormal iNOS-related NO signaling may more readily exert widespread effects across multiple cognitive domains^28^.In the early stages of schizophrenia, NOS-related alterations may reflect dysregulation of neuroregulatory signaling pathways rather than persistent inflammatory activation. This may partly explain why higher iNOS levels within the patient group were associated with better immediate memory performance in the present study^14^. However, this interpretation requires further validation through longitudinal or experimental studies.

A noteworthy finding is that, while the iNOS/TNOS ratio showed some association with certain cognitive domains in correlation analyses, it failed to enter the multivariate regression model as an independent predictor even after controlling for demographic variables and symptom severity. This finding has significant methodological and mechanistic implications. Previous studies have predominantly emphasized the potential role of NOS subtype imbalance—particularly the relative enhancement of inflammation-associated NOS—in the pathophysiology of SCZ^29^. However, the findings of the present study suggest that, at least in the early stages of the disease, the relative changes in the composition of NOS subtypes themselves may not be the key determinants of cognitive impairment. Their apparent association may primarily reflect the influence of overall NOS functional levels or other covariates^30^. In contrast, the evaluation of NO bioavailability from a systemic functional perspective may be more helpful to understand its relationship with cognitive impairment^9,31,32^. The role of the iNOS/TNOS ratio may vary across different disease stages or levels of inflammatory burden, and its potential significance warrants further validation in longitudinal studies or studies with a larger sample size.

Regarding symptom dimension, the TNOS level showed a significant association with the PANSS total score in the multiple regression analysis, a finding that warrants further discussion. This finding suggests that the overall functional state of the NOS system is not only associated with cognitive function but may also partially reflect the overall disease burden of SCZ. Previous studies indicate that the NO signaling pathway participates in the onset and maintenance of psychotic symptoms by regulating neuroinflammatory responses, neurotransmitter system balance, and redox homeostasis^11,33–35^. A key point worth emphasizing is that, in the present study, even after controlling for PANSS total scores, the TNOS level maintained a stable association with multidimensional cognitive function. This suggests that the impact of NOS system dysfunction on cognitive impairment is not mediated by symptom severity alone; rather it may represent a biological pathway that simultaneously spans both symptom and cognitive dimensions^36^. This finding aligns with the current research framework emphasizing cross-dimensional biological mechanisms.

Although peripheral blood NOS levels cannot be directly equated with NO dynamics within the CNS. In addition, TNOS measured in plasma reflects the aggregate enzymatic activity detectable in peripheral circulation and does not distinguish between contributions from different NOS isoforms or tissue sources. Therefore, it should be interpreted as a systemic biochemical marker rather than a direct indicator of NOS activity within the central nervous system^8^. Growing evidence suggests that systemic NO bioavailability, to some extent, can reflect the overall redox and inflammatory state associated with brain function^37,38^. The use of first-episode SCZ samples minimizes confounding effects from chronic disease progression and long-term antipsychotic medication exposure, thereby enhancing the interpretability of peripheral markers in reflecting disease-related biological abnormalities^39^. In addition, the present study incorporated MDA and H_2_O_2_ levels primarily to characterize downstream oxidative stress phenotypes associated with NOS dysfunction, rather than as direct predictors of cognitive function. Elevated lipid peroxidation-related marker levels suggest potential oxidative damage to cell membrane structure and synaptic function, providing contextual support for the involvement of NOS-related pathways in the early pathological processes of SCZ^40^.

Taken together, the oxidative findings observed in the present study do not fully conform to a classical model of generalized oxidative stress upregulation. Instead, the coexistence of reduced iNOS, TNOS, and H₂O₂ levels alongside elevated MDA may reflect a more complex pattern of redox imbalance rather than uniform increases in reactive species. Lipid peroxidation products such as MDA represent downstream markers of oxidative damage to membrane lipids and may accumulate even when circulating levels of certain reactive oxygen species fluctuate or decline^30,41^. In addition, reactive oxygen and nitrogen species operate within a tightly regulated redox network in which alterations in one component can induce compensatory changes in other pathways^42^. For example, decreased circulating H₂O₂ levels may reflect adaptive antioxidant responses or altered metabolic turnover of reactive oxygen species rather than a simple reduction in oxidative activity. Therefore, the present findings may indicate redox dysregulation involving altered nitric oxide signaling and lipid peroxidation processes in early-stage schizophrenia rather than a uniform pro-oxidant shift^43^.

The present study has certain limitations. First, the cross-sectional design restricts causal inference, making it impossible to determine the temporal sequence and causal relationship between NOS dysfunction and cognitive impairment. Second, while peripheral blood NOS-related markers can partially reflect systemic NO bioavailability, they cannot precisely correspond to the dynamic changes in brain-specific NO signaling within the CNS. Another limitation of the present study is the absence of parallel inflammatory markers (such as cytokines or C-reactive protein). Although iNOS is often considered an inflammation-inducible enzyme, its activity can also be influenced by multiple physiological regulatory mechanisms. Without concurrent inflammatory measurements, the present findings cannot be directly interpreted within a broader inflammatory framework. Future studies integrating inflammatory cytokines and NOS-related markers may help clarify the relationship between inflammatory processes and nitric oxide signaling in schizophrenia^5^. Third, the study focused on first-episode SCZ patients, and the findings primarily reflect biological characteristics in the early disease stage. The applicability of these findings to patients with chronic disease or disease relapse requires further validation. Fourth, despite controlling for major demographic variables and symptom severity in the analysis, the influence of other potential confounding factors (such as lifestyle or acute stress states) cannot be entirely ruled out. Future research should integrate longitudinal follow-up design, multimodal neuroimaging, or cerebrospinal fluid-related markers to conduct more in-depth validation of the dynamic relationship between the NOS system and cognitive function. This would enable to further assess its clinical value as a potential predictive indicator or intervention target.

## Conclusion

Patients with first-episode SCZ exhibit significant abnormalities in the functioning of the NOS system. Findings indicate that systemic NOS-related biochemical activity, rather than the proportion of NOS subtypes, is associated with global and multidomain cognitive functions. This association remains significant even after controlling for demographic factors and symptom severity. In contrast, iNOS may primarily influence short-term cognitive processes such as immediate memory. These observations suggest that assessing the NO pathway status at the systemic functional level may be more beneficial than focusing on individual NOS subtypes for understanding the biological mechanisms underlying early cognitive impairment in SCZ. This approach provides a basis for exploring potential biomarkers and intervention targets.

## Supplementary information

Supplementary

## Data Availability

The data supporting the results of this study are available upon request from the
