# Supplementary material for: Abnormalities in nitric oxide synthase system function and their association with cognitive function in first-episode schizophrenia patients
Source: Schizophrenia (Heidelb). 2026 May 12;12(1):63. doi: 10.1038/s41537-026-00764-9 (PMC13389022; doi:10.1038/s41537-026-00764-9)
Supplement: Supplementary file 1 — Supplementary [file 41537_2026_764_MOESM1_ESM.docx]

**Supplementary Materials**

**Supplementary Figures**





**Fig. S1** Correlation between TNOS Levels and PANSS Total and Factor Scores


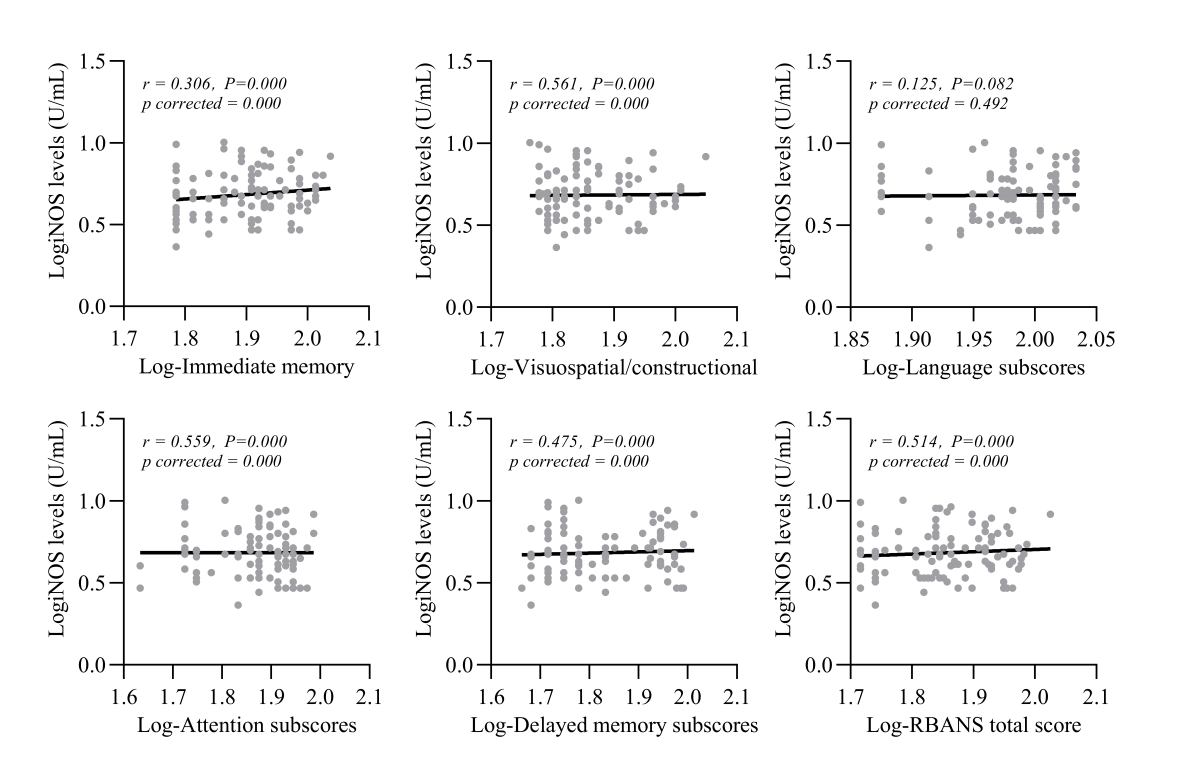


**Fig. S2** Correlation between logiNOS Levels and RBANS Total and Factor Scores


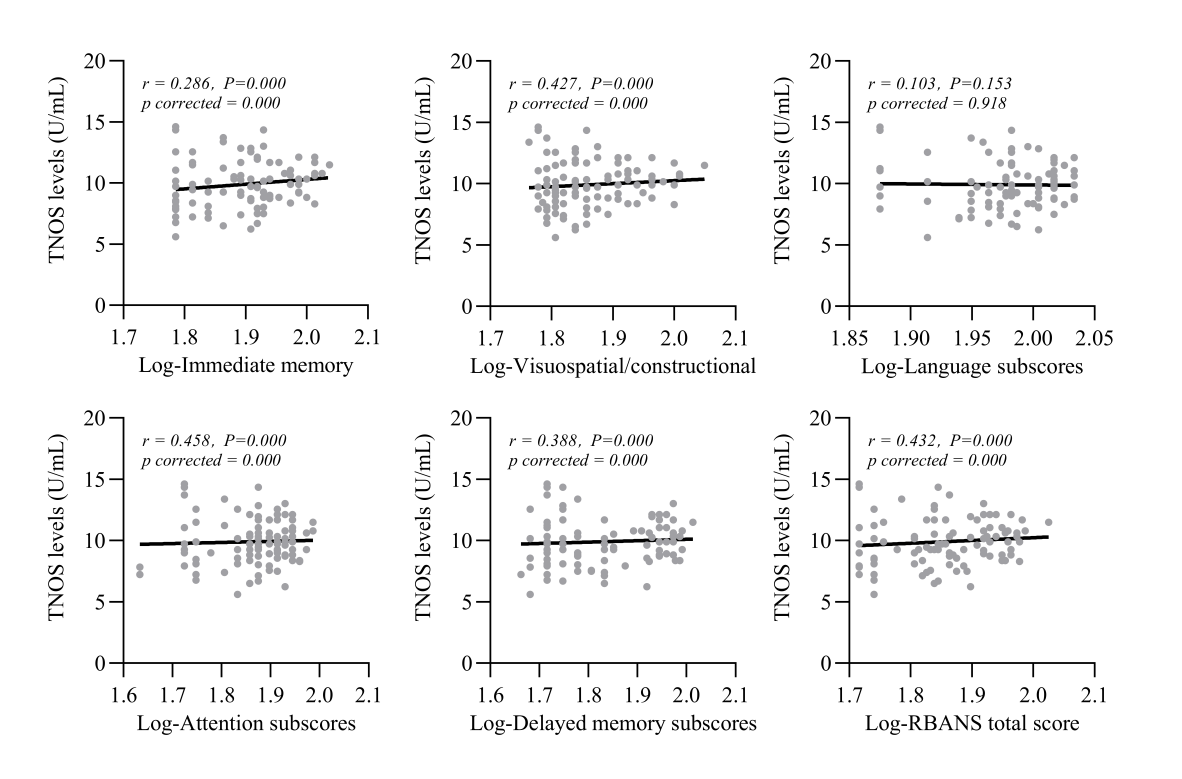


**Fig. S3** Correlation between TNOS Levels and RBANS Total and Factor Scores
